# Seasonal variations in the presentation and growth of thyroid cancer.

**DOI:** 10.1038/bjc.1998.195

**Published:** 1998-04

**Authors:** L. A. Akslen, R. B. Sothern

**Affiliations:** Department of Pathology, The Gade Institute, University of Bergen, Norway.

## Abstract

Seasonal variation has been described in the presentation and growth of several malignant tumours, including cancers of the breast, uterus, uterine cervix, urinary bladder, liver, lymphatic system and skin, although the mechanisms are not known. We herein describe a circannual rhythm for thyroid cancer (total = 2627), with significantly more cases presenting during the late autumn and winter. In a subset of these cases (127 papillary carcinomas), significant seasonal variations with highest values in autumn-winter were found for tumour diameter and proliferation indicators (S- and G2M-phase fractions). These results indicate the likelihood of a seasonal factor (or factors) of importance for the regulation and modification of tumour cell proliferation. When further clarified, this might be of relevance for the planning of diagnostic and therapeutic strategies.


British Journal of Cancer (1998) 77(7), 1174–1179
© 1998 Cancer Research Campaign

### Seasonal variations in the presentation and growth of thyroid cancer

**LA Akslen**[1] **and RB Sothern**[2]

[1]Department of Pathology, The Gade Institute, University of Bergen, Bergen, Norway; [2]Biorhythmometry, College of Biological Sciences, University of Minnesota, St Paul, MN 55108, USA

**Summary** Seasonal variation has been described in the presentation and growth of several malignant tumours, including cancers of the breast, uterus, uterine cervix, urinary bladder, liver, lymphatic system and skin, although the mechanisms are not known. We herein describe a circannual rhythm for thyroid cancer (total = 2627), with significantly more cases presenting during the late autumn and winter. In a subset of these cases (127 papillary carcinomas), significant seasonal variations with highest values in autumn–winter were found for tumour diameter and proliferation indicators (S- and $G_2M$-phase fractions). These results indicate the likelihood of a seasonal factor (or factors) of importance for the regulation and modification of tumour cell proliferation. When further clarified, this might be of relevance for the planning of diagnostic and therapeutic strategies.

**Keywords:** thyroid cancer; season; tumour diameter; proliferation; %S-phase–%$G_2M$-phase

Biological rhythms, especially circadian (24 h) and circannual (12 months), have been found in a wide range of physiological parameters and normal tissues (Aschoff, 1981; Shifrine et al, 1982; Halberg et al, 1983; Lærum et al, 1988; Sothern et al, 1995). Regarding seasonal variations, higher incidence of female breast cancer has been reported for the spring and summer (Cohen et al, 1983; Mason et al, 1985), and circannual contrasts have also been found for other tumours (Newell et al, 1985; Swerdlow, 1985; Hermida and Ayala, 1996). As for the thyroid, some studies have reported seasonal variations in endocrine parameters, with variables such as $T_3$, $T_4$ and thyroid-stimulating hormone (TSH) being higher in the autumn or winter (Halberg et al, 1981; Nicolau et al, 1987; Haus et al, 1988). As seasonal variations might be of potential interest for the detection and management of thyroid carcinomas, we wanted to review this group of tumours, most of which are slowly growing and have a good prognosis.

### MATERIALS AND METHODS

### Patients

During the period 1970–85, 2627 patients with thyroid cancer were reported to the Cancer Registry of Norway. Of these, 10% ($n$ = 263) were surgically treated at the Department of Surgery, Haukeland University Hospital, in the period 1971–85. There were no major differences in the distribution of sex, age and histological types between our hospital cases and cases in the population-based Cancer Registry (Akslen and Myking, 1992). After histological re-examination of the 263 cases for exclusion of benign lesions and subtyping of malignant tumours according to the 1988 WHO criteria, 127 of these cases were found to represent papillary thyroid carcinomas with a diameter greater than 10 mm (microcarcinomas ≤ 10 mm were excluded). These 127 cases were included for further studies of seasonal variations with reference to time (month) of diagnosis, largest diameter of the primary tumour and proliferation indicators (S-phase and $G_2M$-phase fractions). Most of these patients (93%) were treated with total or near-total thyroidectomy. Regarding time of diagnosis, the date of initial histological/cytological diagnosis was used for both the registry ($n$ = 2627) and hospital ($n$ = 127) cases. The dates for the hospital subgroup are identical to those recorded in the Cancer Registry.

The mean age of the 91 female and 36 male patients was 46.0 years. Of the primary tumours (mean size ± s.e. = 29.7 ± 1.61 mm), 41% were completely intrathyroidal, 39% showed invasion into the thyroid gland capsule and 20% revealed major extrathyroidal extension, whereas 47% had lymph node metastases. Regarding histological grade (Akslen, 1993), 28% of these cases were classified as high-grade carcinomas. Distant metastases at diagnosis were present in two cases.

Data concerning regional and distant tumour recurrences and overall survival was recorded. Deaths from causes other than thyroid cancer were treated as censored observations. The median follow-up time was 137 months (maximum 271 months), and no patient was lost to follow-up.

Flow cytometric analysis and estimation of S-phase and $G_2M$-phase fractions were performed on paraffin-embedded tumour material as previously described (Akslen and Varhaug, 1995).

### Data analysis

No differences in overall means for largest tumour diameter, %S-phase or %$G_2M$-phase were found between men and women by simple $t$-test, and data were subsequently combined for analyses. Monthly and seasonal tumour incidence were tested for time effect

*Received 12 September 1997*
*Accepted 20 October 1997*

*Correspondence to:* LA Akslen, Department of Pathology, The Gade Institute, Haukeland University Hospital, N-5021 Bergen, Norway by chi-square ($n$ = 2627 from national registry; $n$ = 127 local cases). Each data set consisting of monthly totals or means was analysed for circannual rhythm by the least-squares fit of a 1-year cosine using the single cosinor procedure (Halberg et al, 1972), which has been adapted to the Macintosh computer (Mojón et al, 1992). In addition, harmonic components (6 months, 4 months) were added to the 1-year cosine model to see if a composite cosine would more accurately describe the true waveform of each data set (Portela et al, 1995), although at this time no biological significance can be ascribed to these additional components. The rhythm characteristics estimated by the cosinor procedure include the mesor (middle value of the fitted cosine representing the rhythm-adjusted mean), the amplitude (half the difference between the minimum and maximum of the fitted cosine) and the acrophase (time of peak value of fitted cosine). A $P$-value for rejection of the zero amplitude (no rhythm) assumption was determined, indicating whether or not the cosine model accounted for a significantly greater proportion of the variability in the time series when compared with the total variability around a flat line (the overall mean). Rhythm detection was considered statistically significant with a $P$-value of ≤0.05. Although the cosinor method involving only a single fitted period may not accurately represent the true characteristics of the actual time-dependent variations if asymmetries exist in a time series, the procedure nevertheless is useful for objectively assessing and quantifying periodicities selected a priori – in our case, the year (Sothern, 1994). Because the mostly serially independent data were collected at unequidistant intervals over a number of years, the single cosinor method was thought to be the only procedure that could provide an objective estimate, not only of the circannual amplitude but also the circannual acrophase, i.e. time of peak value (Klemfuss and Clopton, 1993).

Associations were assessed by Pearson's chi-square test. Univariate survival analysis (product-limit method) was performed using the BMDP-1L program, using the log-rank test for differences between groups. Recurrence-free survival, i.e. the time from diagnosis (in radically treated patients) until the first appearance of regional recurrences or distant spread, and overall patient survival (survival time until death from thyroid cancer) were compared across seasons.

### RESULTS

### Registry cases

The 2627 thyroid cancer cases diagnosed in Norway during 1970–85 showed a highly significant time-effect when incidence was compared across 12 months ($\chi^2$ = 119.3, $P$<0.00001) or four seasons ($\chi^2$ = 35.6, $P$<0.00001), being highest during the last 3-month period October–December (Figure 1A). A 1-year cosine analysis was not significant ($P$ = 0.163; Table 1), but a circannual pattern was prominent with highest values between September and January and fewer patients presenting during February–August, including the months of July and August when many Norwegians take vacation (Figure 1B). The addition of 6 or 4 months to the cosine model did not improve rhythm detection.

### Hospital cases

The number of cases in our hospital series ($n$ = 127 papillary carcinomas) showed a similar pattern of presentation, with 39, 32, 19 and 37 cases occurring in consecutive 3-month periods from January to December ($\chi^2$ for effect of season = 7.65, $P$ = 0.05), with 60% of the cases occurring in the 6-month period between October and March. In these 127 cases, mean tumour diameter was 34.6 for men and 27.8 for women. This difference was not statistically significant ($P$ = 0.13). For all data combined, tumour diameter was greatest in the 3-month period of October–December (mean ± s.e. = 33.5±3.6 mm), this value being 24% higher than in the 3-month period (July–September) with the lowest mean value (27.0±2.6 mm) (Figure 1C and D). Assuming a global form of the thyroid carcinomas, this corresponds to a mean increase in tumour volume of about 90% from lowest to highest average tumour size. There was a range of change (ROC) of 55% between lowest (22.7 mm) and highest (35.2 mm) monthly average tumour size (Table 1). A circannual rhythm in tumour size was detected at $P$ = 0.040 by single cosinor analysis of the 12 monthly means, with an acrophase (time of peak) in the late autumn (December 8; 95% limits: October 22 and January 25) and a double amplitude (representing a predictable range of change) of 30% (Table 1). The addition of 6 or 4 months to the cosine model did not improve rhythm detection.

There was no statistically significant difference between sexes for per cent of cells in S-phase (women = 3.42 ± 0.25%, men = 3.69 ± 0.71%; $P$ = 0.29) or $G_2M$-phase (women = 3.94 ± 0.24%, men = 4.62 ± 0.44%; $P$ = 0.21). These proliferation indicators showed a similar circannual pattern, being highest during the last months of the year (Figure 1E–H). On average, mean S-phase fraction increased by 25% from a winter minimum of 3.04% ± 0.43% to an autumn maximum of 3.81% ±0.43% and mean $G_2M$-phase fraction increased by 30% from its spring minimum of 3.64% ± 0.31% to its fall maximum of 4.75% ± 0.48%. Monthly means ranged from 2.43% ± 0.36% to 4.75% ± 1.98% for S-phase (ROC = 95.4%) and from 2.93% ± 0.35% to 5.21% ± 0.80% for $G_2M$-phase (ROC = 77.5%). Cosinor analysis of monthly means showed a significant circannual rhythm for each proliferative indicator (Table 1; Figure 1E–H), with acrophases in the early autumn for S-phase ($P$ = 0.041, double amplitude = 31%, acrophase = September 18) and late autumn for $G_2M$-phase ($P$ = 0.012, double amplitude = 21%, acrophase = November 3). For illustrative purposes, a composite cosine model consisting of 12+4 months, which seemed to better approximate secondary peaks and troughs for S-phase, and thus the observed waveform in these data, is also shown in Figure 1. The circannual component, however, remained the most prominent, and at this time no biological significance can be ascribed to the 4-month component. Of interest, a 4-month component has recently been used to better describe the circannual pattern for monthly uterine cervical cancer (Hermida and Ayala, 1996).

There was no significant seasonal variation for histological grade, primary tumour extension (pT stage) and presence of lymph node metastases (pN stage) when the 6-month period with maximum tumour diameter (October–March) was compared with the rest of the year. Further, analyses of successive 3-month periods showed no significant seasonal variations for these variables. The disease-free survival, as well as the overall survival of patients presenting during the period October–March (largest tumour diameter) was not different from those presenting during the rest of the year (log-rank test, $P$ = 0.90 and 0.40 respectively).

© Cancer Research Campaign 1998

**Figure 1** Seasonal variation (**A**, **C**, **E** and **G**) of thyroid cancer with respect to number of cases presenting in the Norwegian population (**A** and **B**, *n* = 2627); tumour diameter (**C** and **D**), S-phase fraction (**E** and **F**) and $G_2M$-phase fraction (**G** and **H**) in a subgroup (*n* = 127) of the material (seasonal means ± s.e.m.; some cases were excluded due to lack of information). The graphs in the right column show the corresponding monthly means with fitted 12-month cosine (and with fitted composite 12+4 month cosine when this model helped to describe the observed waveform of the data). Time scale = 3 monthly spans (**A**, **C**, **E** and **G**) or month (**B**, **D**, **F** and **H**). Highest values for each variable were found in autumn–winter, with lowest values in spring–summer.

 *© Cancer Research Campaign 1998*

**Table 1** Circannual rhythm characteristics for thyroid cancer presentation and proliferation indicators observed in Norway, 1970–85

| | | Monthly data characteristics | | | | | | Single cosinor summary for least-squares fit of 1-year cosine | | | | | |
|---|---|---|---|---|---|---|---|---|---|---|---|---|---|
| | | No. of data | | Range of change | | | | | | | | | |
| Variable | Units | Total | Monthly | Low | High | Absolute | Per cent | Mesor ± s.e. | *P* | Amp(A) ± s.e. | (2A)[a] | Acrophase | (95% CL) |
| Incidence | Cases/months | 2627 | 12 | 116 | 282 | 166 | 143% | 222 ± 14 | 0.163 | 37 ± 17 | (33%) | December 19 | (–) |
| Size | Diameter (mm) | 118 | 12 | 22.7 | 35.2 | 12.5 | 55% | 28.8 ± 1.0 | 0.040 | 4.3 ± 1.4 | (30%) | December 8 | (October 22–January 25) |
| S-phase | Per cent | 120 | 12 | 2.43 | 4.75 | 2.32 | 95% | 3.59 ± 0.14 | 0.041 | 0.56 ± 0.17 | (31%) | September 28 | (August 16–November 9) |
| $G_2M$-phase | Per cent | 120 | 12 | 2.93 | 5.21 | 2.28 | 78% | 4.04 ± 0.16 | 0.012 | 0.43 ± 0.10 | (21%) | November 3 | (October 1–December 6) |

[a]Analysis of monthly totals or mean values. Single cosinor, least-squares fit of 1-year cosine to the 12 monthly values. Range of change, difference from lowest to highest value; Mesor, rhythm-adjusted 1-year mean; Amplitude (Amp), 1/2 peak-trough difference of fitted cosine (2A = double amplitude as per cent of Mesor); Acrophase, peak of fitted cosine (in calendar month and day referenced from January 1); CL, confidence limits. *P*-value from non-zero amplitude test.

### DISCUSSION

Our results indicate a significant seasonal variation in the presentation of thyroid cancer. The distribution of 2627 cases reported to the population-based Cancer Registry was highest in the late autumn and winter (September–January), and the hospital subgroup of 127 papillary carcinomas showed a similar pattern. Circannual variation has also been described for breast cancer detection, but most of these studies indicate a peak presentation in the spring and summer (Cohen et al, 1983; Mason et al, 1985; 1990). The maximum periods are different for these tumours, and this could reflect contrasts in tumour cell proliferation and latency period before clinical presentation. Most thyroid carcinomas are slowly growing (Akslen and Varhaug, 1995), whereas breast carcinomas show a higher proliferative activity. Regarding case ascertainment, we cannot exclude some influence of holiday patterns and decreased access to the health system during the summer months. However, this cannot explain the different maximum periods for various cancers. In addition, seasonal variations have been found in a wide range of physiological parameters (Halberg et al, 1983), as well as in the proliferative activity of normal human tissues (Sothern et al, 1995), strongly supporting the presence of an intrinsic biological rhythm.

A significant circannual rhythm in thyroid tumour diameter and cell proliferation was found in the subseries of papillary carcinomas, indicating a high extent of synchronization among individuals since this rhythm could be demonstrated as a group phenomenon. According to the cosinor analysis, there was a difference of 30% between lowest and highest value for tumour diameter, and a corresponding difference of 31% and 21% for S-phase and $G_2M$-phase respectively. For thyroid tumour diameter, there was a maximum period in the last months of the year between September and January, with a peak on December 8. In breast cancer, studies have shown that these tumours were also largest in the autumn (Hartveit et al, 1983; Hartveit, 1992). For thyroid tumour cell proliferation, maximal tumour growth was found in autumn and winter for both S-phase and $G_2M$-phase fractions, and cosinor analysis indicated that the acrophases were located on September 28 for S-phase and November 3 for $G_2M$-phase. Thus, we found almost parallel circannual curves for tumour cell proliferation, tumour diameter and for tumour presentation. Although speculative, this might correspond to a biological sequence: growth signal, tumour cell proliferation, increased tumour diameter and subsequent clinical presentation. Regarding other tumour types, a significant difference during the year has been reported for DNA synthesis (S-phase) in human malignant lymphoma cells (Smaaland et al, 1993). When using data from patients with non-Hodgkin's lymphoma, a significant circannual rhythm was detected with an amplitude of 41% and a peak located on 8 February. Thus, maximum tumour activity was found in the winter, as opposed to the summer for healthy bone marrows and rectal mucosa (Smaaland et al, 1992; Sothern et al, 1995).

Several factors should be considered to explain the seasonal contrasts in presentation and growth of thyroid cancer. Of possible importance, a significant variation with time of the year has been found for TSH, the most important growth factor for thyroid tissue, with peak values in November (Halberg et al, 1981). In breast cancer, hormonal factors have also been discussed, and seasonal differences have been found for oestrogen/progesterone receptors (Holdaway et al, 1990). Seasonal variation of breast cancer presentation further seems to be most pronounced among

© Cancer Research Campaign 1998

women below 50 years of age (Cohen et al, 1983), suggesting a relationship to menstrual status.

Recent studies have indicated an independent relationship between time of presentation and the subsequent prognosis for patients with breast cancer, and those presenting during the spring/summer period might have the best prognosis (Mason et al, 1987; 1990). In our study of thyroid cancer, survival showed no significant variation with season of presentation.

Our findings support the view (Nicolau and Haus, 1992) that seasonal differences in DNA synthesis in normal and neoplastic tissues may be of clinical importance. Blank et al (1992) performed bone marrow biopsies on patients with various malignancies at different clock hours and calendar dates, and found that the mitotic activity revealed a significant circannual rhythm, with an amplitude of 37% and an acrophase occurring on 27 August, a timing nearly identical to that for DNA synthesis found in healthy bone marrows and rectal mucosa (Sothern et al, 1995). Preclinical studies have also shown a circannual tolerance effect for certain tissues after exposure to anti-cancer agents (Lévi et al, 1988; Mormont et al, 1988). Further, myelotoxicity was more severe in winter than summer in patients treated with chemotherapy for ovarian or bladder cancer (Hrushesky, 1982).

In conclusion, our present study adds further evidence to the existence of circannual rhythms in tumour pathobiology, and such results might be important for the timing of treatment regimens (for example dosing time of cytotoxic agents) and other procedures (for example the right season to examine/screen for early signs of malignancy). In addition, time of the year may also be relevant for epidemiological inference, especially when studying the influence of exogenous exposures on tumour development. Our findings on thyroid cancer seasonality suggest the presence of a seasonal factor (or factors) of importance to the regulation of tumour growth, although some influence of vacation patterns is also probable. When further clarified in larger studies and other locales, such data might be of relevance for the planning of diagnostic and therapeutic strategies.

### ACKNOWLEDGEMENTS

This study has been supported by the Norwegian Cancer Society (grant nos 88261/001 and 94070/001). We thank Bendik Nordanger and Jan Solsvik for excellent technical assistance.

### REFERENCES

Akslen LA (1993) Prognostic importance of histologic grading in papillary thyroid carcinoma. *Cancer* **72**: 2680–2685

Akslen LA and Myking AO (1992) Differentiated thyroid carcinomas: the relevance of various pathological features for tumour classification and prediction of tumour progress. *Virchows Arch A* **421**: 17–23

Akslen LA and Varhaug JE (1995) Oncoproteins and tumour progression in papillary thyroid carcinoma. Presence of epidermal growth factor receptor (EGF-R),
*c-erb*B-2 protein, estrogen receptor related protein (p29), p21-*ras* protein and proliferation indicators in relation to tumor recurrences and patient survival. *Cancer* **76**: 1643–1654

Aschoff J (1981) Annual rhythms in man. In *Biological Rhythms. Handbook of Behavioral Neurobiology*, Vol. 6, Aschoff J (ed), pp. 475–487. Plenum Press: New York

Blank MA, Cornélissen G, Neishtadt EL, Kochrev VA, Yakovlev GYa, Haus E, Halberg E and Halberg F (1992) Circadian–circaseptan–circannual mitotic aspects of the bone marrow chronome of patients with malignancy. In *Workshop on Computer Methods on Chronobiology and Chronomedicine* Halberg F and Watanabe H (eds), pp. 245–262. Medical Review: Tokyo

Cohen P, Wax Y and Modan B (1983) Seasonality in the occurrence of breast cancer. *Cancer Res* **43**: 892–896

Halberg F, Johnson EA, Nelson W, Runge W and Sothern RB (1972) Autorhythmometry – procedures for physiologic self-measurement and their analysis. *Physiol Teach* **1**: 1–11

Halberg F, Cornélissen G, Sothern RB, Wallach LA, Halberg E, Ahlgren A, Kuzel M, Radke A, Barbosa J, Goetz F, Buckley J, Mandel J, Schuman L, Haus E, Lakatua D, Sackett L, Berg H, Wendt HW, Kawasaki T, Ueno K, Uezono K, Matsuoka M, Omae T, Tarquini B, Cagnoni M, Garcia Sainz M, Perez Vega E, Wilson D, Griffiths K, Donati L, Tatti B, Vasta M, Locatelli I, Camagna A, Lauro R, Tritsch G and Wetterberg L (1981) International geographic studies of oncological interest on chronobiological variables. In *Neoplasms – Comparative Pathology of Growth in Animals, Plants and Man,* Kaiser H (ed), pp. 553–596. Williams & Wilkins: Baltimore

Halberg F, Lagoguey M and Reinberg A (1983) Human circannual rhythms over a broad spectrum of physiological processes. *Int J Chronobiol* **8**: 225–268

Hartveit F (1992) Breast carcinoma: periodicity in presentation of metastatic tumour growth in the axilla. *Clin Exp Met* **10**: 329–336

Hartveit F, Thoresen S, Tangen M and Halvorsen JF (1983) Variation in histology and oestrogen receptor content in breast carcinoma related to tumour size and time of presentation. *Clin Oncol* **9**: 233–238

Haus E, Nicolau GY, Lakatua D, and Sackett-Lundeen L (1988) Reference values for chronopharmacology. In *Annual Review of Chronopharmacology*, Vol. 4, Reinberg A, Smolensky M and Labrecque G (eds), pp. 333–424. Pergamon Press: Oxford

Hermida RC and Ayala DE (1996) Reproducible and predictable yearly pattern in the incidence of uterine cervical cancer. *Chronobiol Int* **13**: 305–316

Holdaway IM, Mason HB, Marshall RJ, Neave LM and Kay RG (1990) Seasonal change in the concentration of progesterone receptor in breast cancer. *Cancer Res* **50**: 5883–5886

Hrushesky WJM (1982) Bone marrow suppression from doxorubicin and cisdiammine-dichloroplatinum is substantially dependent upon both circadian and circannual state of administration. *Ann NY Acad Sci* **397**: 293–295

Klemfuss H and Clopton PL (1993) Seeking tau: a comparison of six methods. *J Interdisc Cycle Res* **24**: 1–16

Lærum OD, Sletvold O, and Riise T (1988) Circadian and circannual variations of cell cycle distribution in the mouse bone marrow. *Chronobiol Int* **5**: 19–35

Lévi F, Blazsek I and Ferlé-Vidovic (1988) Circadian and seasonal rhythms in murine bone marrow colony-forming cells affect tolerance for the anti-cancer agent 4′-0 tetrahydropyranyladriamycin (THP). *Exp Hematol* **16**: 696–701

Mason BH, Holdaway IM, Mullins PR, Kay RG and Skinner SJ (1985) Seasonal variation in breast cancer detection: correlation with tumour progesterone receptor status. *Breast Cancer Res Treat* **5**: 171–176

Mason BH, Holdaway IM, Skinner SJ, Stewart AW, Kay RG, Neave LM and Anderson J (1987) Association between season of first detection of breast cancer and disease progression. *Breast Cancer Res Treat* **9**: 227–232

Mason BH, Holdaway IM, Stewart AW, Neave LM and Kay RG (1990) Season of initial discovery of tumour as an independent variable predicting survival in breast cancer. *Br J Cancer* **61**: 137–141

Mojòn A, Fernandez JR and Hermida R (1992) ChronoLab: an interactive software package for chronobiologic time series analysis written for the Macintosh computer. *Chronobiol Int* **9**: 403–412

Mormont MC, von Roemeling R, Sothern RB, Berestka JS, Langevin TR, Wick M and Hrushesky WJM (1988) Circadian rhythm and seasonal dependence in the toxicological response of mice to epirubicin. *Invest New Drugs* **6**: 273–283

Newell GR, Lyncy HK, Gibeau JM and Skitz MR (1985) Seasonal diagnosis of Hodgkin's disease among young adults. *J Natl Cancer Inst* **74**: 53–56

Nicolau GY and Haus E (1992) Rhythms in bone marrow cell proliferation. How to apply to the chronotherapy of cancer? *Chronobiol Int* **9**: 393–402

Nicolau GY, Dumitriu L, Plinga L, Petrescu E, Sackett-Lundeen L, Lakatua DJ and Haus E (1987) Circadian and circannual variations of thyroid function in children 11±1.5 years of age with and without endemic goiter. In *Advances in Chronobiology – Part B*, pp. 229–247. Alan R. Liss: New York

Portela A, Cornélissen G, Halberg F, Halberg Francine, Halberg J, Hofman MA, Swaab DF, Ikonomou OC and Stoynev AG (1995) Metachronanalysis of circannual and circasemiannual characteristics of human suprachiasmatic vasopressin-containing neurons. *In Vivo* **9**: 347–358

Shifrine M, Garsd A and Rosenblatt LS (1982) Seasonal variation in immunity of humans. *J Interdisc Cycle Res* **13**: 157–165

Smaaland R, Lærum OD, Sothern RB, Sletvold O, Bjerknes R and Lote K (1992) Colony-forming units – granulocyte/macrophage and DNA synthesis of human bone marrow are circadian stage-dependent and show covariation. *Blood* **79**: 2281–2287

© Cancer Research Campaign 1998

Smaaland R, Lote K, Sothern RB and Lærum OD (1993) DNA synthesis and ploidy in non-Hodgkin's lymphomas demonstrate intrapatient variation depending on circadian stage of cell sampling. *Cancer Res* **53**: 3129–3138

Sothern RB (1994) Circadian and circannual characteristics of blood pressure self-measured for 25 years by a clinically-healthy man. *Chronobiologia* **21**: 7–20

Sothern RB, Smaaland R and Moore JM (1995) Circannual rhythm in DN synthesis (S-Phase) in human bone marrow and rectal mucosa. *Faseb J* **8**: 397–403

Swerdlow AJ (1985) Seasonality of presentation of cutaneous melanoma, squamous cell cancer and basal cell cancer in the Oxford region. *Br J Cancer* **52**: 893–900

© Cancer Research Campaign 1998


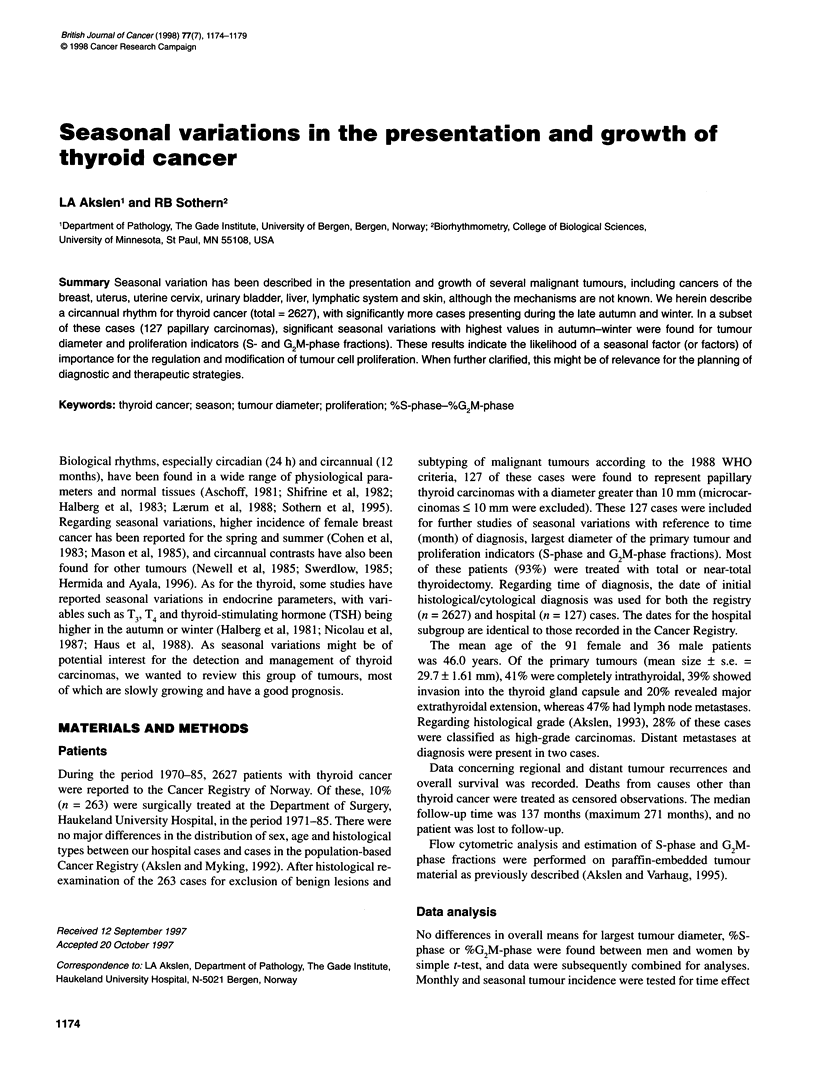

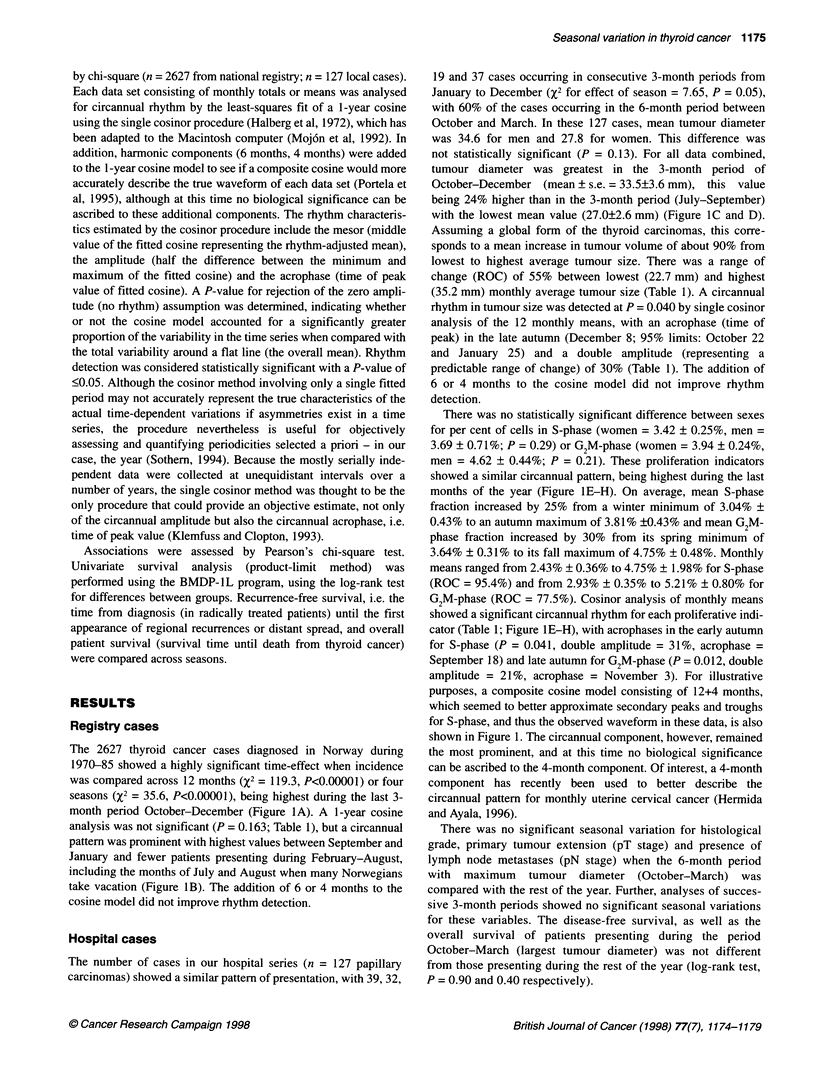

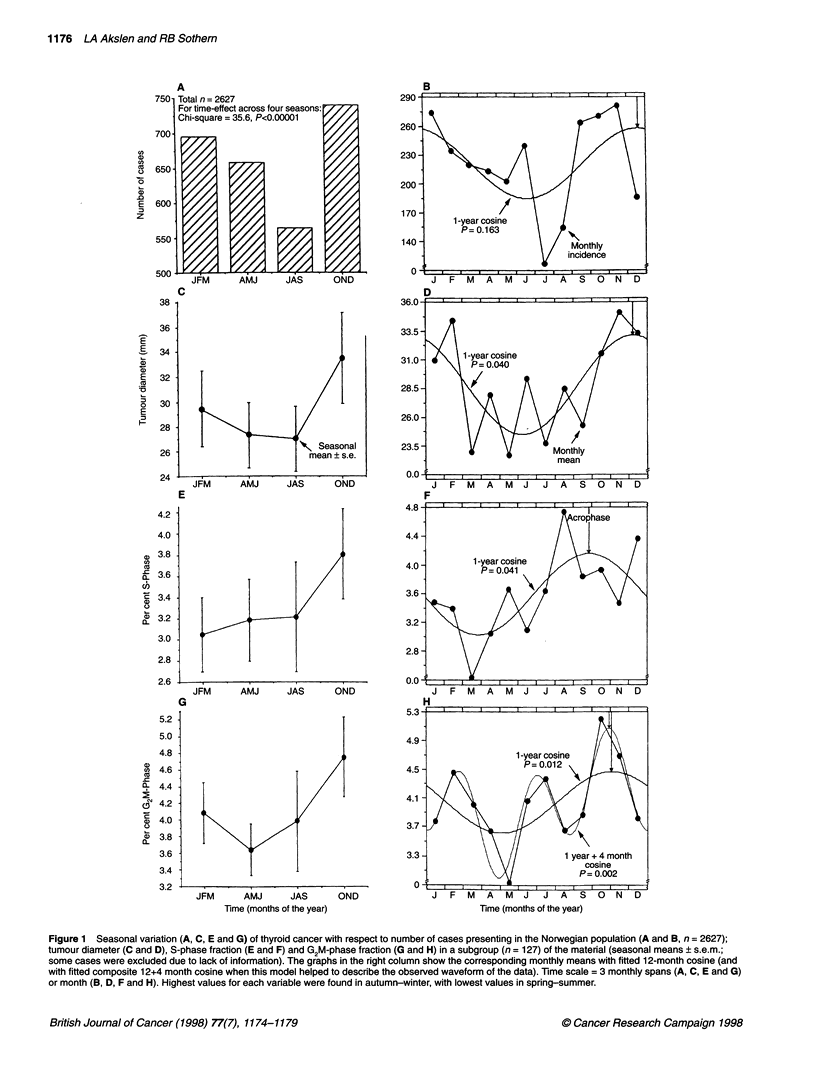

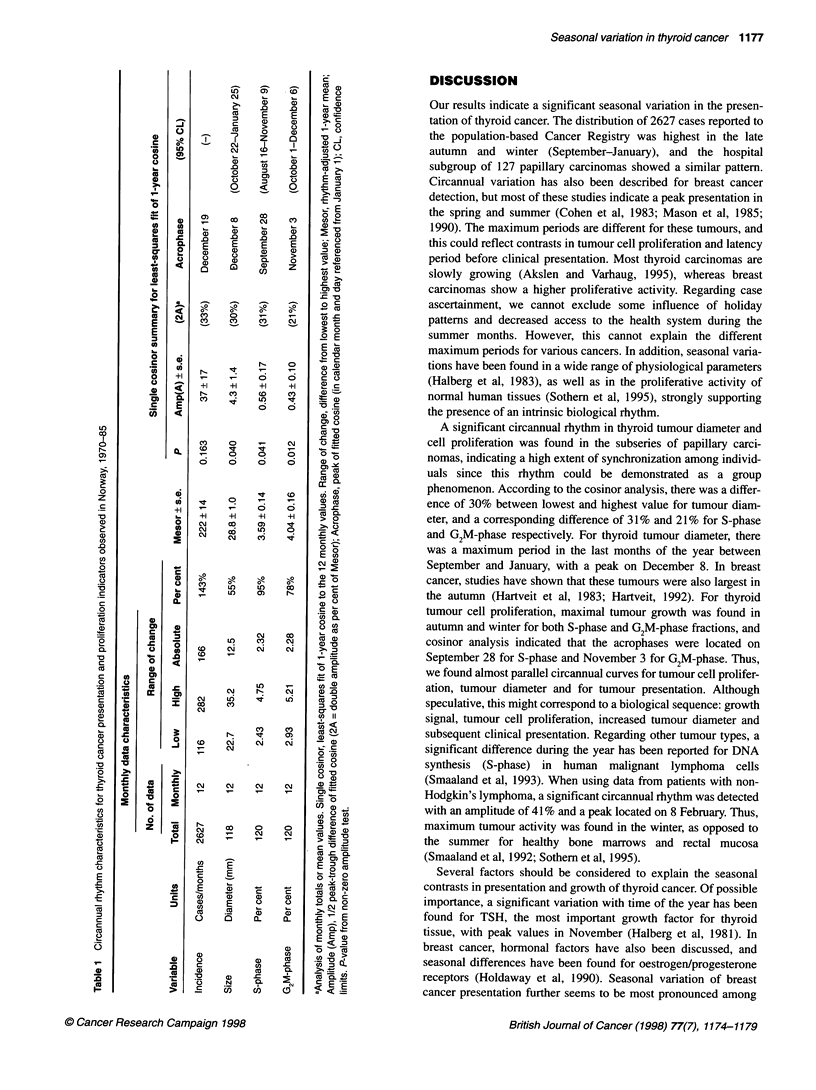

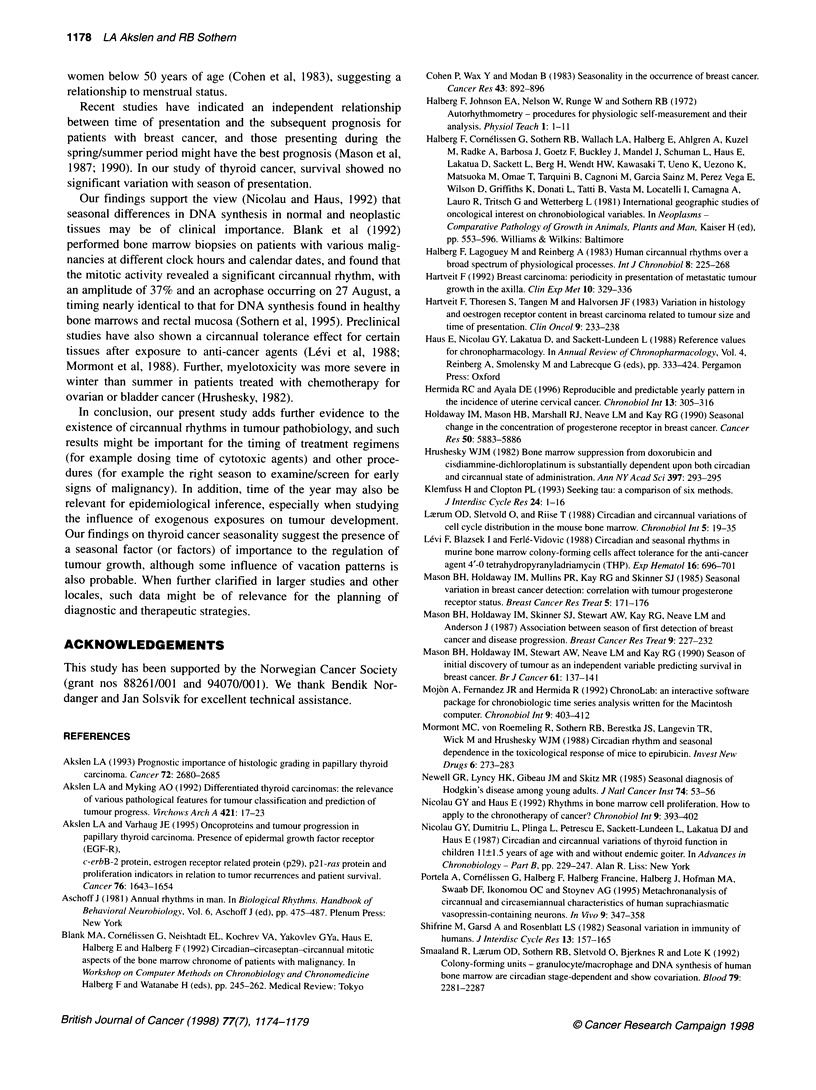

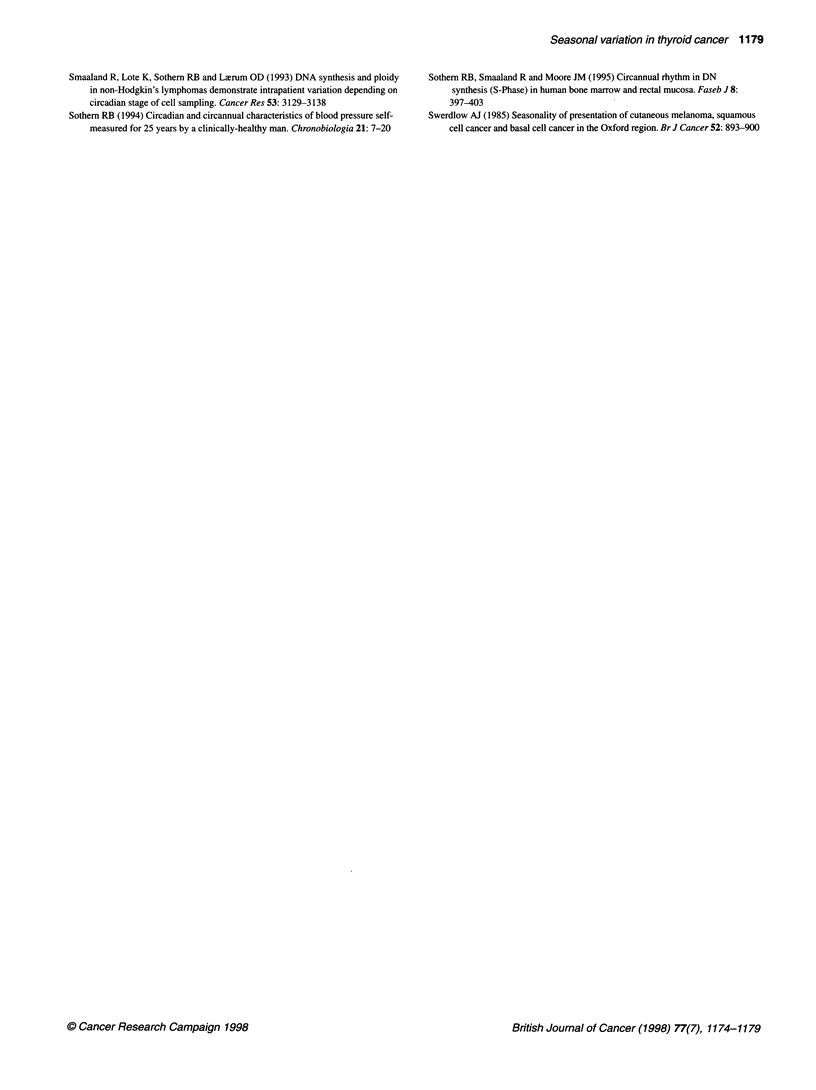


## References

[OCR_00917] Akslen L. A., Myking A. O. (1992). Differentiated thyroid carcinomas: the relevance of various pathological features for tumour classification and prediction of tumour progress.. Virchows Arch A Pathol Anat Histopathol.

[OCR_00913] Akslen L. A. (1993). Prognostic importance of histologic grading in papillary thyroid carcinoma.. Cancer.

[OCR_00922] Akslen L. A., Varhaug J. E. (1995). Oncoproteins and tumor progression in papillary thyroid carcinoma: presence of epidermal growth factor receptor, c-erbB-2 protein, estrogen receptor related protein, p21-ras protein, and proliferation indicators in relation to tumor recurrences and patient survival.. Cancer.

[OCR_00944] Cohen P., Wax Y., Modan B. (1983). Seasonality in the occurrence of breast cancer.. Cancer Res.

[OCR_00967] Halberg F., Lagoguey M., Reinberg A. (1983). Human circannual rhythms over a broad spectrum of physiological processes.. Int J Chronobiol.

[OCR_00971] Hartveit F. (1992). Breast carcinoma: periodicity in presentation of metastatic tumour growth in the axilla.. Clin Exp Metastasis.

[OCR_00975] Hartveit F., Thoresen S., Tangen M., Halvorsen J. F. (1983). Variation in histology and oestrogen receptor content in breast carcinoma related to tumour size and time of presentation.. Clin Oncol.

[OCR_01043] Haus E., Nicolau G. Y. (1992). Rhythms in bone marrow cell proliferation: how to apply to the chronotherapy of cancer?. Chronobiol Int.

[OCR_00986] Hermida R. C., Ayala D. E. (1996). Reproducible and predictable yearly pattern in the incidence of uterine cervical cancer.. Chronobiol Int.

[OCR_00990] Holdaway I. M., Mason B. H., Marshall R. J., Neave L. M., Kay R. G. (1990). Seasonal change in the concentration of progesterone receptor in breast cancer.. Cancer Res.

[OCR_01004] Laerum O. D., Sletvold O., Riise T. (1988). Circadian and circannual variations of cell cycle distribution in the mouse bone marrow.. Chronobiol Int.

[OCR_01007] Lévi F., Blazsek I., Ferlé-Vidovic A. (1988). Circadian and seasonal rhythms in murine bone marrow colony-forming cells affect tolerance for the anticancer agent 4'-O-tetrahydropyranyladriamycin (THP).. Exp Hematol.

[OCR_01012] Mason B. H., Holdaway I. M., Mullins P. R., Kay R. G., Skinner S. J. (1985). Seasonal variation in breast cancer detection: correlation with tumour progesterone receptor status.. Breast Cancer Res Treat.

[OCR_01017] Mason B. H., Holdaway I. M., Skinner S. J., Stewart A. W., Kay R. G., Neave L. M., Anderson J. (1987). Association between season of first detection of breast cancer and disease progression.. Breast Cancer Res Treat.

[OCR_01022] Mason B. H., Holdaway I. M., Stewart A. W., Neave L. M., Kay R. G. (1990). Season of initial discovery of tumour as an independent variable predicting survival in breast cancer.. Br J Cancer.

[OCR_01027] Mojón A., Fernández J. R., Hermida R. C. (1992). Chronolab: an interactive software package for chronobiologic time series analysis written for the Macintosh computer.. Chronobiol Int.

[OCR_01032] Mormont M. C., von Roemeling R., Sothern R. B., Berestka J. S., Langevin T. R., Wick M., Hrushesky W. J. (1988). Circadian rhythm and seasonal dependence in the toxicological response of mice to epirubicin.. Invest New Drugs.

[OCR_01039] Newell G. R., Lynch H. K., Gibeau J. M., Spitz M. R. (1985). Seasonal diagnosis of Hodgkin's disease among young adults.. J Natl Cancer Inst.

[OCR_01047] Nicolau G. Y., Dumitriu L., Plinga L., Petrescu E., Sackett-Lundeen L., Lakatua D. J., Haus E. (1987). Circadian and circannual variations of thyroid function in children 11 +/- 1.5 years of age with and without endemic goiter.. Prog Clin Biol Res.

[OCR_01056] Portela A., Cornélissen G., Halberg F., Halberg F., Halberg J., Hofman M. A., Swaab D. F., Ikonomou O. C., Stoynev A. G. (1995). Metachronanalysis of circannual and circasemiannual characteristics of human suprachiasmatic vasopressin-containing neurons.. In Vivo.

[OCR_01064] Smaaland R., Laerum O. D., Sothern R. B., Sletvold O., Bjerknes R., Lote K. (1992). Colony-forming unit-granulocyte-macrophage and DNA synthesis of human bone marrow are circadian stage-dependent and show covariation.. Blood.

[OCR_01074] Smaaland R., Lote K., Sothern R. B., Laerum O. D. (1993). DNA synthesis and ploidy in non-Hodgkin's lymphomas demonstrate intrapatient variation depending on circadian stage of cell sampling.. Cancer Res.

[OCR_01079] Sothern R. B. (1994). Circadian and circannual characteristics of blood pressure self-measured for 25 years by a clinically-healthy man.. Chronobiologia.

[OCR_01083] Sothern R. B., Smaaland R., Moore J. G. (1995). Circannual rhythm in DNA synthesis (S-phase) in healthy human bone marrow and rectal mucosa.. FASEB J.

[OCR_01088] Swerdlow A. J. (1985). Seasonality of presentation of cutaneous melanoma, squamous cell cancer and basal cell cancer in the Oxford Region.. Br J Cancer.

